# Seeking support: insights into women’s mental health help-seeking behavior in Bangladesh

**DOI:** 10.3389/fgwh.2025.1679141

**Published:** 2025-11-05

**Authors:** Hannah Walter, Maggie E. Craig, Masum Ali, Shahriar Faruque, Sanjib Saha

**Affiliations:** 1 Department of Clinical Sciences (Malmö), Lund University, Lund, Sweden; 2 Nutrition and Health Science, Laney Graduate School, Emory University, Atlanta, GA, United States; 3 Adult Psychiatry, Directorate General of Health, Dhaka, Bangladesh

**Keywords:** Women's mental health, Bangladesh, anxiety, depression, help-seeking behavior, health autonomy, informal support, Demographic and Health Survey

## Abstract

**Introduction:**

Depression and anxiety are leading contributors to the global burden of disease among women yet help-seeking for mental health concerns remains limited in lower-middle-income countries. This study aimed to estimate the prevalence of anxiety, depression, and help-seeking behaviors, and to identify factors associated with the absence of help-seeking among ever-married women of reproductive age in Bangladesh.

**Methods:**

We conducted a cross-sectional analysis using nationally representative data from the 2022 Bangladesh Demographic and Health Survey. Mental health outcomes were assessed using the Generalized Anxiety Disorder-7 and Patient Health Questionnaire-9 screening tools. Help-seeking behavior was defined as any attempt to obtain external support for mental health concerns. Multivariable logistic regression models, accounting for survey design and sampling weights, were used to examine predictors of not seeking help following Behavioral Model of Health Services Use by Andersen and Davidson.

**Results:**

Among 19,987 women aged 15–49 years, 5.1% reported depression, 19.7% reported anxiety, and 20.4% had either condition. Only 20.5% of those with anxiety or depression reported help-seeking, predominantly from informal sources such as family and neighbors. Rural residence, older age, and a professional diagnosis of anxiety were associated with increased help-seeking, while low health autonomy and residence in the Barishal division were linked to lower help-seeking. Formal help-seeking was rare, and significant regional disparities were observed. One in five ever-married women of reproductive age in Bangladesh experiences anxiety or depression, but help-seeking remains low, especially for formal services.

**Discussion:**

Informal networks play a critical role in support. Interventions should address contextual and individual barriers, strengthen social support, and improve access to mental health care, particularly in underserved regions.

## 1 Introduction

Mental health is foundational to overall well-being, enabling individuals to cope with life's stresses, realize their potential, and contribute to their communities. The etiology of mental disorders is complex, influenced by structural, individual, and community-level factors (1). Despite its importance, mental health remains an often neglected component of global health policy and systems, even though research consistently shows strong connections between mental and physical health. Depression and anxiety—the most prevalent mental disorders—are associated with increased risk for chronic diseases, functional impairment, and all-cause mortality (2–7).

Globally, the burden of mental disorders is higher among women than men, a disparity attributed to biological, psychological, and sociocultural factors, including gender roles and life events such as menstruation, pregnancy, and menopause (5, 8–10). In 2021, the global prevalence of anxiety and depression among women aged 15–49 years was approximately 7% and 6%, respectively (8). Lower-middle-income countries (LMICs) are disproportionately affected, with an estimated 82% of all people living with mental disorders residing in LMICs (5, 11).

In Bangladesh, a South Asian LMIC, mental health challenges are pronounced. The National Mental Health Survey 2019 found that 18.7% of adults had at least one mental disorder, with a higher burden among women compared to men (9). Depressive (7.9%) and anxiety disorders (5.4%) are the most common (8, 9), and women in Bangladesh face additional risks due to higher rates of physical and sexual violence, as well as early marriage, which is associated with elevated depressive symptoms among adolescent and adult women (5, 10, 12–14). Despite these challenges, the treatment gap for mental health in Bangladesh is alarmingly high, with about 90% of adults with mental disorders not receiving appropriate care, mainly due to the predominance of depressive and anxiety disorders (9, 15). Access to formal mental health services is further limited by factors including availability, accessibility, and affordability (16–18). Next to formal help-seeking behavior, it is therefore essential to also understand informal help-seeking behavior within familial and friendship dynamics, its enablers and barriers. This distinction is needed to consider both forms of help-seeking in policy design for women's mental health.

In Bangladesh as in many other LMICs, research on mental health and help-seeking behavior especially among women is limited (19, 20). This poses the risk of creating policies and interventions based on assumption about magnitudes, associations and directionalities that differ to higher income contexts. While some studies have been conducted in the South Asia region (21–25), to our knowledge only two quantitative studies on help-seeking were conducted in Bangladesh, focusing on a rural district, and university students (26, 27). The country-representative National Mental Health Survey 2019 described treatment-seeking for mental health; however, they did not assess associations with sociodemographic factors. Help-seeking behavior was conceptualized differently across studies—as intentions, attitudes or reported past behaviors.

Therefore, understanding formal and informal help-seeking behavior and the predictors of help-seeking behavior among women in Bangladesh is critical to addressing the treatment gap and improving mental health outcomes. The Sustainable Development Goals explicitly address mental health, underscoring its relevance to broader public health and development objectives (3, 28). This study utilizes data from the Bangladesh Demographic and Health Survey (BDHS) 2022 to assess the prevalence of anxiety, depression, and help-seeking behaviors among ever-married women of reproductive age (15–49 years), and to identify factors associated with the absence of help-seeking for mental health problems in this population.

## 2 Materials and methods

### 2.1 Data source

**Figure 1:**
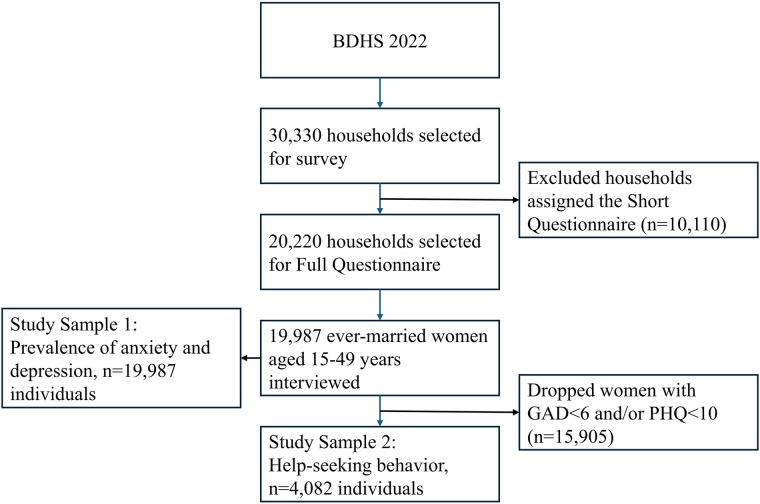
Flow chart presenting the sample selection for the analysis of prevalences of anxiety and depression, and help-seeking behavior using BDHS 2022 data. BHDS, Bangladesh Demographic and health survey; GAD, generalized anxiety Disorder-7; PHQ, patient health questionnaire-9.

The study utilized secondary data from the BDHS 2022, a survey that is nationally representative. The survey employed a two-stage stratified sampling technique to guarantee the representation of the whole population residing in non-institutional households across Bangladesh. The initial phase entailed the selection of 675 enumeration units (clusters) using a probability proportional to size sampling method, succeeded by a comprehensive household listing inside each chosen unit. In the second phase, 45 households from each enumeration area were selected (*n* = 30,330) and ever-married women aged 15 to 49 were interviewed (19,987, response rate: 98.9%). Ever-married women were those women currently married or married previously, but widowed, divorced or separated at the time of data collection. See Figure 1 for further information on the sample selection. The analytical sample was restricted to the sub-sample of ever-married women aged 15 to 49 years who were screened for anxiety and depression. More information on sampling can be obtained from the official survey report (29).

### 2.2 Conceptual model

**Figure 2:**
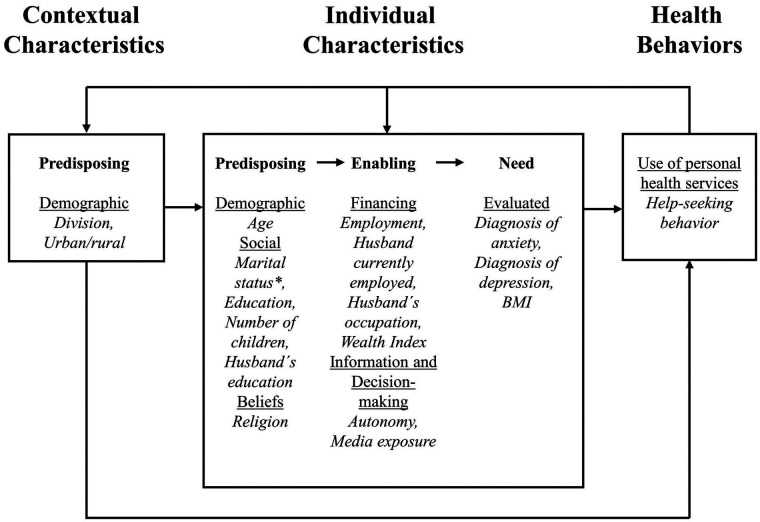
Behavioral model of health services use (30) adapted to seeking help for anxiety and/or depression.

The study was guided by the Behavioral Model of Health Services Use by Andersen and Davidson (30), which conceptualizes healthcare access as influenced by both individual and contextual factors (Figure 2). These are categorized into predisposing (demographics, social structure, health beliefs), enabling (resources, policies, organizational factors), and need (perceived or evaluated need for care) components. Predisposing and need factors are generally less mutable, whereas enabling factors are more amenable to policy intervention. Based on this framework and prior research, relevant variables from the BDHS 2022 were selected for analysis.

### 2.3 Variables

#### 2.3.1 Outcome variables

This study examined four outcome variables: anxiety, depression, any anxiety or depression, and help-seeking behavior. For the mental health outcomes of anxiety and depression, cut-offs were selected based on the reviewed literature (22, 23). This increased interpretability of results but did not allow to look at the full spectrum of mental health and may yield different results than clinical diagnoses.

*Anxiety* was measured using the Generalized Anxiety Disorder-7 (GAD-7) scale (31), a 7-item questionnaire assessing symptoms over the past two weeks, scored from 0 (never) to 3 (always). Scores were classified as mild (0–5), moderate (6–14), and severe (15–49), with a score of 6 or higher indicating anxiety (binary: yes/no).

*Depression* was assessed using the Patient Health Questionnaire-9 (PHQ-9) (32), comprising nine items scored 0–3 over the past two weeks. Severity was categorized as minimal/no symptoms (0–4), mild (5–9), moderate (10–14), moderately severe (15–19), and severe (20–27). A score of 10 or higher indicated depression (binary: yes/no).

*Any anxiety or depression* was defined as the presence of either anxiety or depression, or both, vs. neither.

*Help-seeking behavior* was defined as the attempt to obtain external assistance for mental health concerns. In this study, help-seeking behavior was assessed by self-report of past help-seeking (yes/no). While this simplification was necessary for appropriate analyses and interpretation, the findings do not account for the number of help sources, time spent seeking or receiving help, and success of the help-seeking practice. The sources of help were categorized: formal (doctor/medical personnel, social service organization, social worker, community health worker/fieldworker, NGO) and informal (religious leader, spouse/partner, family member, friend, neighbor).

#### 2.3.2 Independent variables

Variables were classified according to the Andersen model (30) (Figure 2). For contextual predisposing factors, place of residence and division were included. Place of residence was a binary variable (urban, rural), while the variable division was categorized according to the eight administrative divisions in Bangladesh: Barishal, Chattogram, Dhaka, Khulna, Mymensingh, Rajshahi, Rangpur and Sylhet.

Individual characteristics were grouped according to predisposing, enabling and need factors as portrayed by the Andersen model. Predisposing factors were age (15–24, 25–34, 35–49 years), marital status (currently married, widowed/divorced/separated), number of children (none, 1–2, ≥3), woman's and husband's highest education level (no education, primary, secondary, higher), and religion (Islam, others). Education was treated as a predisposing factor due to its low mutability.

Enabling factors were current employment (yes/no), Wealth Index (poorest, poorer, middle, richer, richest), husband's employment status (worked last 7 days: yes/no), husband's occupation (non-manual/manual), women's autonomy in health decisions (respondent alone, joint, husband/other), and media exposure (yes/no). In the BDHS, wealth quintiles were calculated with principal component analysis that each comprises 20% of the population and consider owned consumer goods and housing characteristics. Husband's occupation was categorized as non-manual (not working, professional/technical/managerial, clerical, sales) or manual (agricultural, household/domestic, services, skilled/unskilled manual), following previous BDHS research. Media exposure included radio, newspaper, magazine, internet, or TV (any vs. none).

Need factors were body mass index (BMI) and prior diagnoses of anxiety or depression. BMI was dichotomized using the World Health Organization's (WHO's) Asian criteria: normal (18.5–22.9) and underweight/overweight/obese (<18.5 or >22.9) (33). Diagnoses were based on prior professional assessment by healthcare providers.

### 2.4 Statistical analysis

Descriptive statistics were presented as frequencies and percentages. Associations between independent and dependent variables were assessed using Pearson's chi-square tests. Bivariate relationships were examined with simple logistic regression, using absence of help-seeking as the reference. Multivariate logistic regression included all significant factors from bivariate analyses, reporting odds ratios (OR), adjusted odds ratios (aOR), 95% confidence intervals (CI), and *p*-values. Statistical significance was set at α < 0.05. The statistical software automatically excluded collinear variables from adjusted models adjusted models. Analyses were restricted to participants with anxiety (GAD-7 ≥ 6) and/or depression (PHQ-9 ≥ 10). Sensitivity analyses can be obtained in the Supplementary Material. We performed unadjusted and adjusted logistic regressions for the absence of informal help-seeking behavior among the subsample of women seeking only informal help to determine whether this largest source of help was consistent with our main analysis. Additionally, we tested for interaction of selected predictors (highest level of education and autonomy over health decisions) to assess whether associations differed by values of other variables (Supplementary Material). Data were analyzed using Stata/SE 18.0 accounted for sampling weights and survey design to ensure national representativeness (29).

### 2.5 Ethical considerations

The study utilized publicly available secondary data from the 2022 BDHS. Ethical approval was obtained from the ICF Institutional Review Board and the Bangladesh Medical Research Council (29). All data were anonymized and analyzed at the population level. Respondents with a PHQ-9 score ≥10 or any suicidal ideation were offered mental health services.

## 3 Results

### 3.1 Prevalence of anxiety, depression and any depression or anxiety

The sample consisted of 19,987 ever-married women aged 15 to 49 years. Within the sample, 5.1% (*n* = 1,026) were identified as experiencing depression. Anxiety was reported by 19.7% (*n* = 3,925) of the participants. The prevalence of either mental condition was 20.4% (*n* = 4,082) among the participating women.

### 3.2 Prevalence of help-seeking behavior and predictors of not seeking help

Among women with any anxiety or depression (*n* = 4,082), 20.5% (*n* = 836) reported help-seeking behavior. Chi-square analyses indicated that division, current employment, women's autonomy, media exposure, diagnosis of anxiety, and diagnosis of depression were significantly associated with the absence of help-seeking behavior, while no individual predisposing factors showed significant associations (Table 1). In the adjusted regression analysis, several contextual and individual factors were significant predictors of help-seeking (Table 2). A forest plot (Supplementary Figure S1, Supplementary Material) presents an overview of the adjusted regression analysis results. Contrary to common expectations, women residing in rural areas had significantly lower odds of not seeking help compared to those in urban areas (aOR: 0.68; 95% CI: 0.50–0.94). Compared to Barishal, all divisions except Sylhet had lower odds of absence of help-seeking, with Khulna showing the lowest odds (aOR: 0.42; 95% CI: 0.24–0.74). Older age was associated with increased help-seeking, as women aged 35–49 years had significantly lower odds of not seeking help than those aged 15–24 years (aOR: 0.63; 95% CI: 0.41–0.97). Regarding enabling factors, joint health decision-making with a husband was associated with 60% higher odds of not seeking help compared to independent decision-making by the woman (aOR: 1.60; 95% CI: 1.13–2.25). Finally, a professional diagnosis of anxiety was linked to a 67% reduction in the odds of not seeking help (aOR: 0.33; 95% CI: 0.21–0.51).

**Table 1:** Sociodemographic and health-related characteristics by help-seeking behavior among ever-married women aged 15–49 years in Bangladesh.

Variable	No help-seeking (*n* = 3,246, 79.5%)	Help-seeking (*n* = 836, 20.5%)	*p*-value*
Contextual characteristics			
Predisposing			
Place of residence			
Urban	1,106 (34.1%)	278 (33.3%)	0.079
Rural	2,140 (65.9%)	558 (66.7%)	
Division			
Barishal	363 (11.2%)	57 (6.8%)	0.001
Chattogram	509 (15.7%)	148 (17.7%)	
Dhaka	436 (13.4%)	93 (11.1%)	
Khulna	395 (12.2%)	172 (20.6%)	
Mymensingh	311 (9.6%)	66 (7.9%)	
Rajshahi	366 (11.3%)	109 (13.0%)	
Rangpur	478 (14.7%)	129 (15.4%)	
Sylhet	388 (12.0%)	62 (7.4%)	
Individual characteristics			
Predisposing			
Age			
15–24 years	555 (17.1%)	125 (15.0%)	0.168
25–35 years	1,099 (33.9%)	269 (32.2%)	
35–49 years	1,592 (49.0%)	442 (52.9%)	
Marital status			
Currently married	2,935 (90.4%)	767 (91.7%)	0.362
Widowed/divorced/separated	311 (9.6%)	69 (8.3%)	
Highest level of education			
No education	629 (19.4%)	123 (14.7%)	0.050
Primary	949 (29.2%)	241 (28.8%)	
Secondary	1,339 (41.3%)	378 (45.2%)	
Higher	329 (10.1%)	94 (11.2%)	
Number of children			
None	299 (9.2%)	71 (8.5%)	0.147
1–2	1,744 (53.7%)	447 (53.5%)	
3 or more	1,203 (37.1%)	318 (38.0%)	
Husband's highest level of education			
No education	835 (28.5%)	186 (24.3%)	0.345
Primary	847 (28.9%)	217 (28.3%)	
Secondary	864 (29.5%)	248 (32.4%)	
Higher	383 (13.1%)	115 (15.0%)	
Religion			
Islam	2,987 (92.0%)	764 (91.4%)	0.172
Others	259 (8.0%)	72 (8.6%)	
Enabling			
Current employment			
No	2,212 (68.1%)	516 (61.7%)	0.008
Yes	1,034 (31.9%)	320 (38.3%)	
Wealth Index			
Poorest	676 (20.8%)	153 (18.3%)	0.861
Poorer	717 (22.1%)	167 (20.0%)	
Middle	629 (19.4%)	170 (20.3%)	
Richer	656 (20.2%)	184 (22.0%)	
Richest	568 (17.5%)	162 (19.4%)	
Husband currently employed			
No	294 (10.0%)	88 (11.5%)	0.605
Yes	2,636 (90.0%)	676 (88.5%)	
Husband's occupation			
Non-manual	840 (28.7%)	235 (30.8%)	0.922
Manual	2,088 (71.3%)	528 (69.2%)	
Woman's autonomy (decision over own health)			
Wife alone	418 (14.2%)	178 (23.2%)	0.000
Husband/wife together	1,846 (62.9%)	368 (48.0%)	
Husband alone/other	671 (22.9%)	221 (28.8%)	
Media exposure			
No	1,250 (38.5%)	251 (30.0%)	0.024
Yes	1,996 (61.5%)	585 (70.0%)	
Need			
Diagnosis of anxiety			
No	2,842 (87.6%)	565 (67.6%)	0.000
Yes	404 (12.4%)	271 (32.4%)	
Diagnosis of depression			
No	2,971 (91.5%)	649 (77.6%)	0.000
Yes	275 (8.5%)	187 (22.4%)	
BMI			
Normal	573 (34.7%)	131 (29.9%)	0.084
Underweight/overweight/obese	1,078 (65.3%)	308 (70.2%)	

* Pearson's chi-square test *p* < 0.05.

**Table 2:** Details of univariate and multivariate logistic regressions for the association of sociodemographic and health-related characteristics with the absence of help-seeking behavior among ever-married women aged 15–49 years in Bangladesh with any depression or anxiety.

Variable	OR	95% CI	*p*-value	aOR*	95% CI	*p*-value
Contextual characteristics						
Predisposing						
Place of Residence						
Urban	1.00			1.00		
Rural	0.81	0.64–1.03	0.088	0.68	0.50–0.94	0.018
Division						
Barishal	1.00			1.00		
Chattogram	0.52	0.32–0.85	0.009	0.53	0.30–0.91	0.022
Dhaka	0.72	0.43–1.23	0.229	0.51	0.28–0.95	0.033
Khulna	0.36	0.22–0.59	0.000	0.42	0.24–0.74	0.003
Mymensingh	0.73	0.45–1.20	0.216	0.51	0.28–0.94	0.030
Rajshahi	0.56	0.33–0.94	0.028	0.54	0.29–0.99	0.046
Rangpur	0.54	0.34–0.88	0.012	0.51	0.29–0.88	0.017
Sylhet	0.87	0.50–1.50	0.605	0.68	0.35–1.32	0.252
Individual characteristics						
Predisposing						
Age						
15–24 yrs	1.00			1.00		
25–34 yrs	0.89	0.69–1.16	0.392	0.87	0.58–1.29	0.481
35–49 yrs	0.80	0.63–1.01	0.065	0.63	0.41–0.97	0.038
Marital status						
Currently married	1.00			was omitted from multivariable logistic regression due to collinearity		
Widowed/divorced/separated	1.16	0.83–1.61	0.379			
Highest level of education						
No education	1.00			1.00		
Primary	0.82	0.64–1.06	0.139	0.78	0.52–1.17	0.229
Secondary	0.72	0.56–0.91	0.007	0.69	0.45–1.05	0.080
Higher	0.80	0.57–1.13	0.199	0.72	0.38–1.36	0.307
Number of children						
None	1.00			1.00		
1–2	0.83	0.61–1.13	0.228	0.71	0.42–1.18	0.182
3 or more	0.74	0.54–1.01	0.058	0.66	0.37–1.19	0.166
Husband's highest level of education						
No education	1.00			1.00		
Primary	0.89	0.71–1.11	0.286	1.07	0.77–1.51	0.676
Secondary	0.85	0.68–1.07	0.171	0.86	0.61–1.22	0.403
Higher	0.78	0.59–1.05	0.097	0.60	0.35–1.02	0.060
Religion						
Islam	1.00			1.00		
Others	0.73	0.48–1.11	0.140	0.74	0.43–1.25	0.260
Enabling						
Current employment						
No	1.00			1.00		
Yes	0.78	0.65–0.93	0.006	0.82	0.62–1.08	0.151
Wealth index						
Poorest	1.00			1.00		
Poorer	0.97	0.76–1.25	0.820	1.23	0.83–1.82	0.303
Middle	0.87	0.65–1.16	0.338	1.00	0.66–1.52	0.998
Richer	0.91	0.68–1.22	0.532	1.16	0.74–1.81	0.516
Richest	0.97	0.72–1.32	0.860	1.35	0.84–2.17	0.221
Husband currently employed						
No	1.00			1.00		
Yes	1.08	0.82–1.42	0.599	0.90	0.61–1.32	0.581
Husband's occupation						
Non-manual	1.00			1.00		
Manual	1.01	0.83–1.22	0.922	1.12	0.84–1.50	0.444
Woman's autonomy (decision over own health)						
Wife alone	1.00			1.00		
Husband/wife together	2.23	1.74–2.87	0.000	1.60	1.13–2.25	0.008
Husband alone/other	1.38	1.07–1.79	0.015	1.05	0.73–1.53	0.787
Media exposure						
No	1.00			1.00		
Yes	0.79	0.65–0.97	0.026	0.97	0.72–1.31	0.837
Need						
Diagnosis of anxiety						
No	1.00			1.00		
Yes	0.27	0.21–0.35	0.000	0.33	0.21–0.51	0.000
Diagnosis of depression						
No	1.00			1.00		
Yes	0.28	0.21–0.37	0.000	0.97	0.58–1.62	0.914
BMI						
Normal	1.00			1.00		
Underweight/overweight/obese	0.80	0.62–1.04	0.091	0.82	0.63–1.07	0.152

OR, odds ratio; aOR, adjusted odds ratio; CI, confidence interval.

* aOR adjusted by all factors included in the Andersen model (see Figure 2): Place of Residence, division, age, highest level of education, number of children, husband's highest level of education, religion, current employment, wealth index, husband's current employment, husband's occupation, woman's autonomy, media exposure, BMI, diagnosis of anxiety, diagnosis of depression.

### 3.3 Sources for help

**Figure 3:**
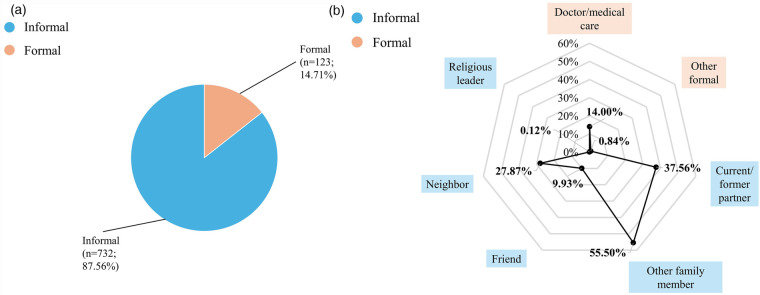
Help-Seeking with any anxiety or depression among ever-married women aged 15–49 years in Bangladesh (*n* = 836), BDHS 2022. The denominator for percentages entails everyone with any depression or anxiety seeking help. Because multiple sources of help could be selected by respondents, percentages add up to more than 100%. Other formal help-seeking sources include social service organizations, social workers, community health workers and NGOs. **(a)** Formal and informal help-seeking among ever-married women aged 15–49 years in Bangladesh with any anxiety or depression (*n* = 836), BDHS 2022. **(b)** Sources of help sought by ever-married women aged 15–49 years in Bangladesh with any anxiety or depression (*n* = 836), BDHS 2022.

**Figure 4:**
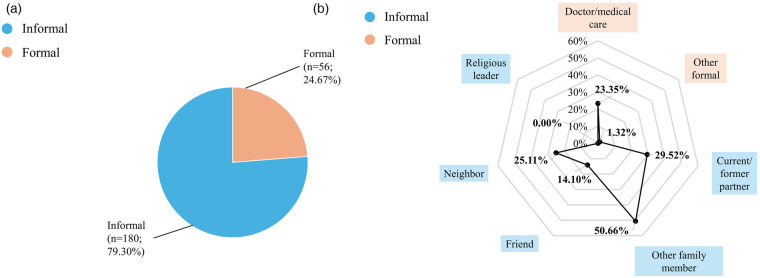
Help-Seeking with depression among ever-married women aged 15–49 years in Bangladesh (*n* = 227), BDHS 2022. The denominator for percentages entails everyone with anxiety seeking help. Because multiple sources of help could be selected by respondents, percentages add up to more than 100%. Other formal help-seeking sources include social service organizations, social workers, community health workers and NGOs. **(a)** Formal and informal help-seeking among ever-married women aged 15–49 years in Bangladesh with depression (*n* = 227), BHDS 2022. **(b)** Sources of help sought by ever-married women aged 15–49 years in Bangladesh with depression (*n* = 227), BDHS 2022.

**Figure 5:**
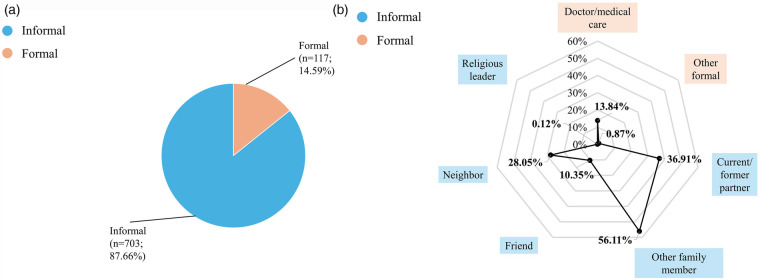
Help-Seeking with anxiety among ever-married women aged 15–49 years in Bangladesh (*n* = 802), BDHS 2022. The denominator for percentages entails everyone with depression seeking help. Because multiple sources of help could be selected by respondents, percentages add up to more than 100%. Other formal help-seeking sources include social service organizations, social workers, community health workers and NGOs. **(a)** Formal and informal help-seeking among ever-married women aged 15–49 years in Bangladesh anxiety (*n* = 802), BDHS 2022. **(b)** Sources of help sought by ever-married women aged 15–49 years in Bangladesh with anxiety (*n* = 802), BDHS 2022.

Among women with any anxiety or depression who sought help, the majority (87.6%, *n* = 732) (Figures 3–5) relied on informal sources. Only 3.8% (*n* = 28) accessed both informal and formal help, and 22.8% of those who sought formal help also used informal sources. Doctors or medical care personnel were the most common formal source (14.0%, *n* = 117), with formal help-seeking more frequent among women with depression (23.4%) than those with anxiety (13.8%). Other formal sources, such as social service organizations, social workers, community health workers, fieldworkers, and NGOs, were rarely utilized (0.8%, *n* = 7).

Family members other than a current or former husband/partner were the most frequent source of help overall (55.5%, *n* = 464) (Figures 3–5). Current or former husbands/partners (37.6%, *n* = 314) and neighbors (27.9%, *n* = 233) were also common sources, while friends (9.9%, *n* = 83) and religious leaders (0.1%, *n* = 1) were less frequently approached. No major differences in help-seeking sources were observed between women with anxiety and those with depression.

## 4 Discussion

### 4.1 Prevalence of anxiety, depression and any depression or anxiety

The prevalence of depression was lower than previously reported in the National Mental Health Survey 2019 for adult women in Bangladesh (7.9%) (9) and the Global Burden of Disease Study 2021 estimates for women aged 15–49 years (8.3%) (8). In contrast, the prevalence of anxiety disorders among our sample (19.6%) was substantially higher than the rates reported in these studies, which found 5.4% (9) for adult women and 5.5% for women aged 15–49 years (8). This discrepancy may be attributed to a stricter multi-stage case identification including diagnoses by trained research psychiatrists for the National Mental Health Survey or reliance on older source data from 1996, 1997 and 2000 in the Global Burden of Disease Study. Furthermore, the use of distinct assessment instruments and varying cutoff points complicates direct comparisons of prevalence rates across studies.

Notably, the overall prevalence of any depression or anxiety in our sample (20.4%) closely aligns with the National Mental Health Survey 2019 finding of 21.5% for mental disorders among women (9). However, that study utilized the Self Reporting Questionnaire to screen for 24 psychotic and non-psychotic mental disorders whereas we only included depression and anxiety. This highlights the heterogeneity in indicator measurement. Our results are consistent with data from a nationally representative survey in Nepal, which reported similar prevalence rates for depression (5.4%) and anxiety (21.9%) among women (34). These findings underscore the importance of methodological consistency and up-to-date data sources when interpreting mental health prevalence estimates across different contexts.

### 4.2 Help-seeking behavior

We found that help-seeking among women with depression or anxiety in Bangladesh was generally low, consistent with findings from other South Asian countries and LMICs (22, 23, 35). Compared to Nepal, help-seeking was approximately 10% lower in our sample, though differences in sample composition—such as the inclusion of never-married women and those with very mild symptoms in the Nepalese study—may account for some of this disparity (23).

The majority of women who sought help did so through informal channels, primarily family members, friends, and neighbors. This pattern aligns with previous research from Bangladesh and the broader South Asian context, which highlights the central role of families and local networks in providing support for mental health concerns (9, 23, 35–37). The WHO's Situational Assessment on Mental Health in Bangladesh also emphasized the strength of family networks as a resource for mental health support (15). Notably, women in rural areas were more likely to seek help than those in urban settings, a finding that contrasts with some earlier studies attributing higher urban help-seeking rates to better healthcare infrastructure (17). This may be explained by a greater reliance on informal support in rural areas (38), where higher physical proximity and stronger social cohesion facilitate access to help, especially for women engaged primarily in domestic work (36). Women in rural Bangladesh may have lower odds of not seeking help compared to urban women because rural communities often foster stronger social connectivity and support networks that promote interpersonal communications, frequently unrecognized as a kind of help-seeking behavior (39). Though family structures in Bangladesh are changing, women in rural Bangladesh usually still live in joint families which facilitates contact with family members and neighbors (40). Women living in urban areas are more likely to work outside the home which might reduce opportunities for social contacts.

Contrary to some reports, very few women sought help from religious leaders (9, 15), despite the significant role faith and traditional healers play in general health care in Bangladesh. Mental disorders in the country are often attributed to supernatural causes which may lead to a preference for spiritual healers rather than medical professionals (9, 17, 41). However, stigma surrounding mental health may discourage women from seeking help from these sources for mental health issues.

Formal help-seeking, particularly among women with anxiety, was much less common than informal help-seeking (9, 23), and when it occurred, it was mainly from medical professionals such as physicians. Barriers to formal help-seeking can be both person-centric—such as stigma, low health literacy, and cultural beliefs—and system-centric, including a lack of trained professionals, poor service quality, and limited resource distribution (42). These challenges contribute to persistent treatment gaps and low utilization of formal mental health services (9, 18, 36, 38).

Geographical disparities were evident, with women in Barishal division having significantly higher odds of not seeking help compared to other regions. Bangladesh is ranked one of the most climate vulnerable areas globally which affects women's mental health negatively (43, 44). Barishal division is among the most vulnerable regions to climate change in Bangladesh, especially due to its coastal location. Its unique geography exposes it to more frequent cyclones, tidal surges, flooding, salinity intrusion, and water logging compared to other divisions with potential consequences on healthcare accessibility and help-seeking rates (45). This finding is further exacerbated by potential higher levels of anxiety and depression in Barishal division, connected to climate change-related extreme weather events, in particular floodings, which were previously identified for adolescents (46). However, the overall low levels of formal help-seeking suggest that regional differences may also reflect variations in informal support networks or other contextual factors.

The conceptual model highlighted the importance of predisposing contextual elements in influencing help-seeking behavior. In analyzing environmental and systemic factors, it is essential to consider the present public health expenditure on mental health. Notwithstanding the reported high prevalence rates of two significant mental diseases and the Lancet Commission's recommendation to commit a minimum of 5% of health expenditures to mental health (3), in 2020, just approximately 0.05% of total public health expenditure in Bangladesh was dedicated to mental health (15). This highlights a significant disparity between high demand and constrained human, financial, and physical resources, particularly prevalent in LMICs (35). Mental health is currently excluded from social insurance plans, rendering it unaffordable for numerous Bangladeshi women (17, 38). The restricted funding for mental health may lead to low rates of formal help-seeking and significant disparities across divisions (47).

In the fully adjusted models, the only individual predisposing factor that significantly predicted lower odds of not seeking help was being beyond the age of 34. In Nepal, help-seeking was less prevalent among older age groups; however, no significant associations were tested (22), nor was any variation in help-seeking across age groups detected (34). Previous studies indicate that stigma, which hinders help-seeking, is more pronounced among older individuals (41). Mental disorders are being connected to magic and religion and people living with mental disorders are perceived as dangerous, having personal weaknesses, causing family shame and having failed spiritually/morally (41). Nevertheless, the global literature regarding the correlation between age and the propensity to seek mental health assistance remains inconclusive (48). Hypotheses supporting higher help-seeking among older age groups may be increased time availability due to older children, broader social networks among family, friends, and neighbors, or comorbidities that heighten the necessity for overall health assistance.

Low health autonomy was identified as a predictor of the absence of help-seeking behavior. Individuals who collaborate with their spouses in health decision-making or who are entirely uninvolved may encounter various obstacles when contemplating addressing stigmatized mental health issues (49). Jafree et al. (49) point out, that even educated and working women in Bangladesh live in a system, were making autonomous health decisions might make them “fear being shunned, stigmatized, and labeled a “rebel” for assuming health rights independently” (49, p. 56). This shows the interplay of different barriers to help-seeking such as autonomy, stigma and gender dynamics (41). Enhancing social support networks and fostering self-awareness may facilitate increased help-seeking behaviors for mental health issues (42).

Diagnoses of mental disorders can serve as both motivators for seeking assistance and outcomes of the help-seeking process. The bidirectional relationship necessitates careful interpretation of the strong association between a diagnosis of anxiety and help-seeking behavior. It underscores the significance of diagnoses in the process of seeking help for mental health issues. In Bangladesh, individuals must pursue formal medical assistance to obtain a diagnosis. Nonetheless, a limited percentage of individuals with mental health issues seek assistance from qualified professionals capable of making diagnoses (36). This is also associated with the limited availability of human resources. In 2021, the WHO estimated that Bangladesh had 0.16 psychiatrists, 0.4 specialized mental health nurses, and 0.34 psychologists per 100,000 people (15). The integration of mental health services into primary care and the provision of training in mental health care at this level may be significantly pertinent.

Overall, these findings underscore the need for interventions targeting both contextual and individual barriers to mental health care. Effective strategies should build on existing social support networks, reduce stigma, and increase awareness about mental health and available services among both the general population and healthcare providers. Structural changes, including increased financing and integration of mental health into community-level care, are essential, especially in underserved regions such as Barishal. Ensuring that health service structures can reach women in remote areas and address mental health needs is vital for reducing the treatment gap and improving outcomes for women across Bangladesh (50).

### 4.3 Strengths & limitations

This study utilized cross-sectional data, precluding causal inferences between exposures and mental disorders or help-seeking behavior (51). Given the temporal relationship between mental health problems and help-seeking, there is a risk of sample selection bias. By including only women with current anxiety or depression, the study may have excluded those who previously experienced these conditions, sought help, and recovered, potentially underestimating the effectiveness of certain help sources.

The use of existing secondary data introduced further limitations. Symptom data were collected through interviewer-administered surveys, and stigma surrounding mental health may have led to underreporting, resulting in underestimated prevalence rates (16). Differences in cultural understanding and interpretation of mental health could also affect disclosure and complicate cross-country comparisons (5). Although the BDHS employed validated instruments (GAD-7 and PHQ-9), these tools are screening measures and do not substitute for clinical diagnosis; thus, reported cases should be considered probable rather than confirmed disorders.

Help-seeking behavior was assessed based on self-reported past actions and sources. The dataset did not include information on help-seeking intentions, types of assistance, number of help sources, or time frames, nor did it capture self-help behaviors—areas that warrant further investigation in future research (52, 53).

The variables analyzed were primarily individual-level factors, reflecting data availability. However, broader societal and political determinants such as stigma and mental health literacy also play a critical role in mental health and need to be explored to increase explanatory power of study results and to ultimately inform resource allocation and policy development (44). The selection of variables does not capture the full spectrum of predictors for help-seeking but highlights key factors and groups requiring attention. Comorbidities such as thyroid disease, diabetes and hypertension were excluded due to limited data and lack of statistical significance, as their inclusion would have substantially reduced the analytic sample.

This study is aimed to observe Bangladeshi women's mental-health help seeking behavior using BDHS 2022 survey data, but that survey only considered depression and anxiety, ignoring other common mental-health issues like psychosis, obsessive compulsive disorder, bipolar disorder, post-partum blue, stress disorders, somatic symptom disorders etc. This may limit the overall picture of mental-health help-seeking behavior of Bangladeshi women.

Despite these limitations, the study achieved its objectives, providing new insights into the prevalence of anxiety and depression, as well as help-seeking behavior among ever-married women of reproductive age in Bangladesh. The use of reliable, nationally representative data and validated instruments supports the robustness of the findings. While results may be generalizable to similar populations in South Asia, contextual factors—including physical, social, and political environments—should be considered. The conceptual model employed underscored the importance of both contextual and individual determinants of anxiety, depression, and help-seeking behavior.

## 5 Conclusion

This study indicated that approximately 20% of women in Bangladesh experienced depression or anxiety. The likelihood of not seeking help was greatest among women lacking a formal anxiety diagnosis, those residing in the Barishal division, and younger women. Approximately 20% of women experiencing anxiety or depression pursued assistance for their mental health. Informal sources of assistance, such as family and neighbors, were more prevalent than formal support systems. To elucidate these differences and formulate policies and interventions aimed at enhancing mental health in Bangladesh at both structural and societal levels, it is essential to consider additional socio-cultural factors.

## Data Availability

Publicly available datasets were analyzed in this study. This data can be found here: https://www.dhsprogram.com/Countries/Country-Main.cfm?ctry_id=1&;c=Bangladesh&;Country=Bangladesh&;cn=&;r=4.
