# Supplementary material for: Seeking support: insights into women’s mental health help-seeking behavior in Bangladesh
Source: Front Glob Womens Health. 2025 Nov 5;6:1679141. doi: 10.3389/fgwh.2025.1679141 (PMC12627062; doi:10.3389/fgwh.2025.1679141)
Supplement: Supplementary file 1 [file Datasheet1.pdf]

Supplementary Material

1 Predictors of Informal Help-Seeking

For sensitivity analyses, bivariate and adjusted logistic regressions with the outcome variable absence of informal help-seeking were performed (see Table S1).

The results largely corresponded with the predictors of overall help-seeking (see Table 2). This includes that Barishal division had higher odds for an absence of informal help-seeking behavior compared to all other other divisions. Women from rural areas had significantly lower odds to seek help in general but also informally compared to those living in urban areas (aOR: 1.70; 95% CI: 0.50-0.97). Women with lower autonomy over health decisions had higher odds to not seek informal help compared to those with independent health decision-making. This was significant for women who made health decisions together with their husband (aOR: 2.17; 95% CI: 1.56-3.03). In contrast to the predictor analysis of overall help-seeking, no individual predisposing factors were associated with informal help-seeking in the multivariable logistic regression. A diagnosis of anxiety was not associated with significantly lower odds for an absence of informal help-seeking which might be explained by the bidirectional relationship between formal help-seeking and getting a formal diagnosis which was omitted in this analysis.

These supplementary results confirm and support the results of the main analysis of help-seeking behavior and show the relevance of contextual and individual enabling factors for (informal) help-seeking.

Table S1: Details of univariate and multivariate logistic regressions for the association of sociodemographic and health-related characteristics with the absence of informal help-seeking behavior among ever-married women aged 15-49 years in Bangladesh with any depression or anxiety

	OR	95% CI	p-value	aOR*	95% CI	p-value
**Contextual**						
Predisposing						
Division						
Barishal	1.00			1.00		
Chattogram	0.47	0.27–0.84	0.010	0.47	0.26–0.86	0.014
Dhaka	0.68	0.37–1.24	0.206	0.45	0.23–0.86	0.016
Khulna	0.31	0.18–0.54	0.000	0.32	0.17–0.59	0.000
Mymensingh	0.63	0.36–1.12	0.115	0.47	0.24–0.91	0.024
Rajshahi	0.54	0.29–0.99	0.047	0.47	0.24–0.91	0.026
Rangpur	0.56	0.32–1.00	0.050	0.56	0.30–1.07	0.079
Sylhet	0.79	0.43–1.47	0.463	0.72	0.35–1.48	0.368

Place of Residence						
Urban	1.00			1.00		
Rural	0.80	0.61-1.04	0.094	0.70	0.50-0.97	0.035
**Individual characteristics**						
Predisposing						
Age						
15-24 yrs	1.00			1.00		
25-34 yrs	0.90	0.69-1.18	0.461	0.97	0.65-1.43	0.860
35-49 yrs	0.90	0.70-1.15	0.402	0.89	0.57-1.39	0.606
Marital status						
Currently married	1.00			was omitted from multivariable logistic regression due to collinearity		
Widowed/divorced/separated	1.28	0.90-1.83	0.172			
Highest level of education						
No education	1.00			1.00		
Primary	0.80	0.62–1.05	0.104	0.86	0.56-1.34	0.86
Secondary	0.75	0.58–0.97	0.027	0.78	0.50-1.23	0.78
Higher	0.82	0.57–1.17	0.272	0.75	0.38-1.50	0.75
Number of children						
None	1.00			1.00		
1-2	0.83	0.60-1.15	0.252	0.67	0.40-1.12	0.129
3 or more	0.74	0.53-1.03	0.072	0.56	0.31-1.03	0.064
Husband´s highest level of education						
No education	1.00			1.00		
Primary	0.89	0.71-1.12	0.331	1.17	0.82-1.66	0.388
Secondary	0.88	0.70-1.11	0.291	0.93	0.65-1.33	0.703
Higher	0.89	0.65-1.23	0.485	0.74	0.42-1.30	0.293
Religion						
Islam	1.00			1.00		
Others	0.66	0.43-1.02	0.060	0.67	0.39-1.15	0.146

Enabling						
Current employment						
No	1.00			1.00		
Yes	0.76	0.62-0.93	0.007	0.82	0.62-1.10	0.180
Wealth index						
Poorest	1.00			1.00		
Poorer	1.01	0.78-1.31	0.950	1.29	0.85-1.95	0.230
Middle	0.83	0.61-1.12	0.222	0.93	0.60-1.45	0.752
Richer	0.99	0.72-1.36	0.933	1.23	0.77-1.97	0.387
Richest	1.08	0.77-1.51	0.661	1.56	0.94-2.59	0.084
Husband currently employed						
No	1.00			1.00		
Yes	1.10	0.83-1.46	0.509	0.93	0.62-1.39	0.726
Husband´s occupation						
Non-manual	1.00			1.00		
Manual	0.99	0.82-1.21	0.954	1.23	0.92-1.65	0.167
Woman´s autonomy (decision over own health)						
Wife alone	1.00			1.00		
Husband/wife together	2.70	2.11-3.46	0.000	2.17	1.56-3.03	0.000
Husband alone/other	1.62	1.25-2.10	0.000	1.33	0.92-1.93	0.126
Media exposure						
No	1.00			1.00		
Yes	0.85	0.68-1.06	0.138	0.98	0.72-1.33	0.903
Need						
Diagnosis of depression						
No	1.00			1.00		
Yes	0.48	0.36-0.63	0.000	0.86	0.51-1.44	0.568
Diagnosis of anxiety						
No	1.00			1.00		
Yes	0.46	0.36-0.58	0.000	0.65	0.42-1.01	0.054

BMI						
Normal	1.00			1.00		
Underweight/overweight/obese	0.80	0.61-1.04	0.095	0.78	0.59-1.03	0.083

*aOR adjusted by all factors included in the Andersen model (see Figure 1): Place of Residence, division, age, highest level of education, number of children, husband´s highest level of education, religion, current employment, wealth index, husband´s current employment, husband´s occupation, woman´s autonomy, media exposure, BMI, diagnosis of anxiety, diagnosis of depression.

OR, Odds Ratio; aOR, adjusted Odds Ratio; CI, Confidence Interval.

2 Interaction of Autonomy and Education on Help-Seeking Behavior

We tested whether the association between women´s autonomy over health decisions and the absence of help-seeking behavior varied by highest level of education by adding an interaction term in the logistic regression model. It was not statistically significant ($p>0.05$), indicating that the relationship between autonomy and help-seeking did not differ across education levels. Therefore, the interaction term was not included in the final model and only the main effects of the predictors were interpreted. This result highlights that education and autonomy are two related but distinct concepts that are separately connected to help-seeking behavior.

3 Associations of sociodemographic and health-related characteristics with the absence of help-seeking behavior

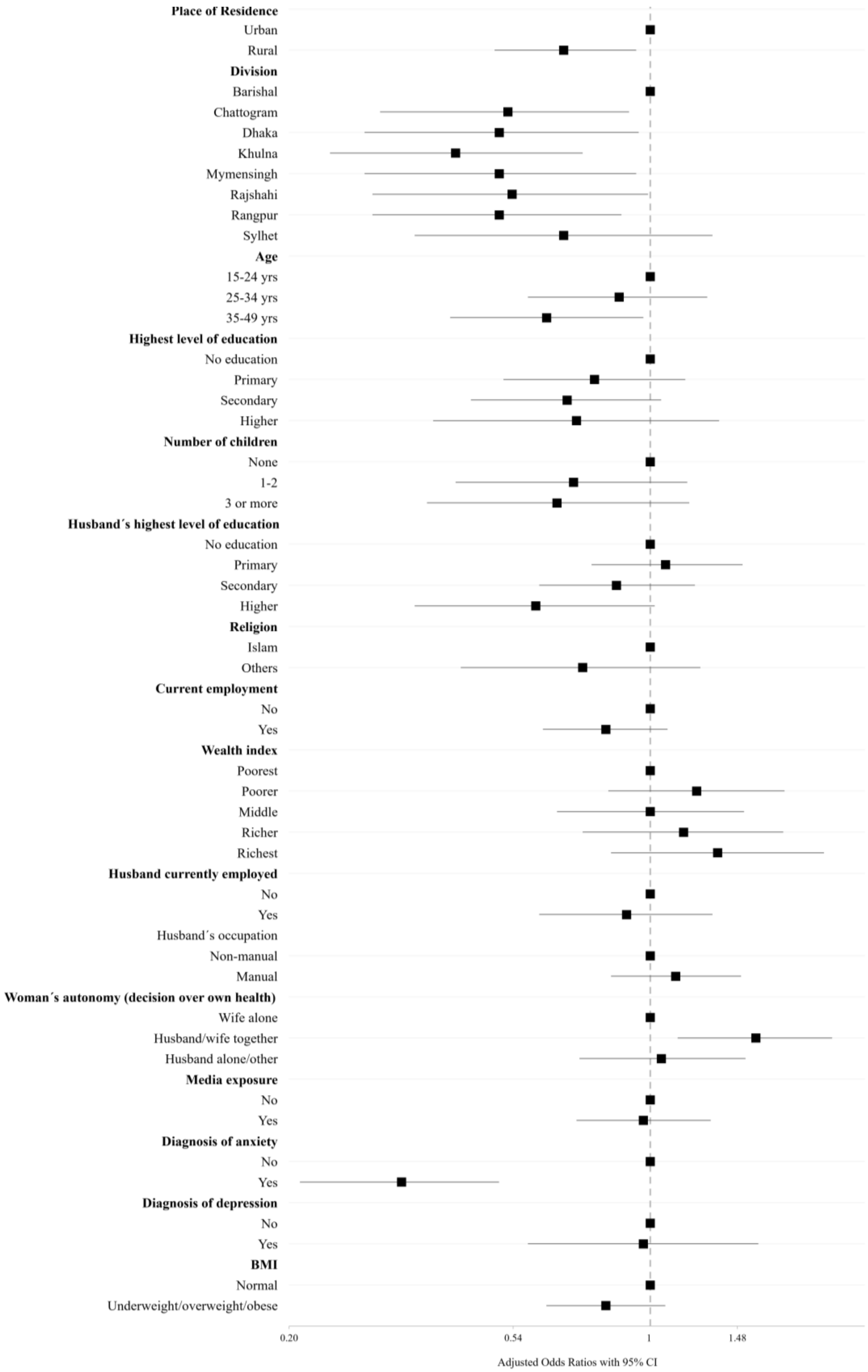

Figure S1: Adjusted Odds Ratios presenting the associations of sociodemographic and health-related characteristics with the absence of help-seeking behavior among ever-married women aged 15-49 years in Bangladesh with any depression or anxiety.

Odds Ratios adjusted by all factors included in the Andersen model: Place of Residence, division, age, highest level of education, number of children, husband´s highest level of education, religion, current employment, wealth index, husband´s current employment, husband´s occupation, woman´s autonomy, media exposure, BMI, diagnosis of anxiety, diagnosis of depression.
